# Innovative Approaches to 3D Printing of PA12 Forearm Orthoses: A Comprehensive Analysis of Mechanical Properties and Production Efficiency

**DOI:** 10.3390/ma17030663

**Published:** 2024-01-29

**Authors:** Andrzej Zakręcki, Jacek Cieślik, Anna Bazan, Paweł Turek

**Affiliations:** 1 Department of Manufacturing Systems, Faculty of Mechanical Engineering and Robotics, AGH University of Science and Technology in Cracow, 30-059 Cracow, Poland; cieslik@agh.edu.pl; 2 Mediprintic sp. z o.o., 39-300 Mielec, Poland; 3 Department of Manufacturing Techniques and Automation, Faculty of Mechanical Engineering and Aeronautics, Rzeszow University of Technology, 35-959 Rzeszow, Poland; abazan@prz.edu.pl (A.B.); pturek@prz.edu.pl (P.T.)

**Keywords:** additive manufacturing, 3D printing, forearm orthosis, polyamide powders, PA12, mechanical properties, SLS, HP MJF, production capacity

## Abstract

This research paper aims to explore the mechanical characteristics of polyamide PA12 (PA12) as a 3D material printed utilizing Selective Laser Sintering (SLS) and HP MultiJet Fusion (HP MJF) technologies in order to design and manufacture forearm orthoses. The study assessed the flowability of the materials used and compared the mechanical performance of PA12 with each other using tensile, flexure, and impact tests in five different fabrication orientations: X, Y, Z, tilted 45° XZ, and tilted 45° YZ. The results of the study provide, firstly—the data for testing the quality of the applied polyamide powder blend and, secondly—the data for the design of the orthosis geometry from the aspect of its strength parameters and the safety of construction. The mechanical parameters of SLS specimens had less variation than MJF specimens in a given orientation. The difference in tensile strength between the 3D printing technologies tested was 1.8%, and flexural strength was 4.7%. A process analysis of the forearm orthoses revealed that the HP MJF 5200 system had a higher weekly production capacity than the EOS P396 in a production variance based on obtaining maximum strength parameters and a variance based on maximizing economic efficiency. The results suggest that medical device manufacturers can use additive manufacturing technologies to produce prototypes and small-batch parts for medical applications. This paper pioneers using 3D printing technology with Powder Bed Fusion (PBF) methods in designing and manufacturing forearm orthoses as a low- to medium-volume product. The applied solution addresses the problem of medical device manufacturers with regard to the analysis of production costs and mechanical properties when using 3D printing for certified medical devices.

## 1. Introduction

Additive Manufacturing (AM) techniques are among the fastest-growing technologies to make even the most geometrically complex models [1,2]. Nowadays, many publications report concern models made by additive manufacturing techniques from polymeric materials used in medicine [3]. The most common applications of polymeric materials are the manufacture of surgical templates or instruments [4,5,6], implants [7,8], and scaffolds [9,10]. Concerning the fact that not only prototypes but often functional models are produced using additive manufacturing technologies, quality requirements are imposed on them related to the assessment of, among other things, mechanical properties [11,12,13], dimensional and geometry accuracy [14,15], and surface roughness [16,17,18]. These properties are closely related to the applied printing parameters, e.g., the applied print layer thickness, the model’s orientation in the 3D printer space, or the model fill density [11,12]. The parameters also affect the manufacturing costs of the model, which is also an important research topic [19,20].

As a result of continuous improvements in the mechanical and performance properties of polymeric materials, their use in the 3D printing process of orthoses, among others, is currently being expanded [21,22,23,24]. For orthoses, it is essential to use the correct type of material to stabilize the joint and maintain a constant temperature around it [24,25,26,27,28,29,30,31,32,33]. Additionally, it is crucial to ensure that the orthosis provides airflow and moisture wicking. For the additive manufacturing of orthoses, the most common materials used are Acrylonitrile Butadiene Styrene (ABS) [29], Polyethylene Terephthalate Glycol (PETG) [34], Polylactic Acid (PLA) [35] or composites [30]. However, these materials are now replaced by polyamide PA12 [29,31]. This is due to this material’s high mechanical, thermal, and fatigue strength [27,30]. In addition, this material is resistant to less aggressive chemicals. This material is also highly hygroscopic, quickly absorbing water from the environment [33,36]. A significant feature of this material is its biocompatibility according to ISO 10993-1, and it is also approved for food contact according to EU Directive 2002/72/EC (excluding alcoholic products) [37,38]. An essential feature that orthosis should fulfill also concerns the aesthetics of its manufacture. The PA12 material offers many possibilities for finishing parts, such as polishing, dyeing, lacquering, powder coating, or gluing the products.

Most commonly, models made from PA12 material are produced using Multi Jet Fusion (MJF) [39,40] and Selective Laser Sintering (SLS) [41,42,43,44,45] technologies. A vital aspect of the mechanical properties obtained may also relate to how successive print layers are fused. In the case of the MJF method, the powder bed in the machine is heated uniformly using a thermal head. Then, the precision print head applies two types of agents to support the printing process. The first agent is dispensed in the model areas, and its properties multiply the absorption of thermal radiation. The second is applied at the outer contours of the parts to facilitate the separation of the unmelted powder [46]. In the case of SLS technology, a laser is used to scan and sinter each layer [39]. Due to the differences in bonding of the print layers, an important aspect is to carry out strength tests. The literature has mainly presented tensile tests on samples made of PA12 material [47,48,49,50] and only in specific orientations [51,52].

Taking into account the literature review concerning the production of forearm orthoses [53,54], research was mainly conducted on models made using the Material Extrusion (MEX) methods with the materials ABS [35,55], PLA [35], PA12 [35], high-impact polystyrene (HIPS) [35] and PLA-CaCO_3_ [30]. Some authors have also extended their research by analyzing the Finite Element Method (FEM) and designed models of orthoses [35,53]. Research is also being introduced into making personalized designs for forearm orthoses using SLS technology. They are designed according to the Design for Additive Manufacturing (DFAM) methodology, considering lattice structure and topology optimization solutions on desktop machines, such as Sinterit LISA 1.5 (Sinterit sp. z o.o., Cracow, Poland) and EOS P395 (EOS GmbH, Krailling, Germany) industrial systems [56,57]. However, there needs to be more research on the broader field of manufacturing forearm orthoses made of nylon by SLS and MJF. A separate issue concerns the evaluation of the manufacturability of the MJF and SLS methods in the context of producing models of forearm orthoses from PA12 material. To date, research on this aspect has not been addressed extensively. Particular attention needs to be paid to, among other things, the maximum number of components to be placed in the working chamber. In addition, as the process of layer-by-layer curing is different in MJF and SLS technology, it is necessary to assess fabrication and cooling times. It is also necessary to pay attention to the start-up time of the 3D printer and material acquisition costs.

Currently, in the healthcare market, some solutions are using 3D printing using Powder Bed Fusion (PBF) methods for the production of orthopedic products, including ankle foot orthoses [58], prosthetic sockets [59], and foot orthotics [60]. On the other hand, for this moment, clinical research is being conducted into personalized orthoses made by 3D printing technologies like the Fused Filament Fabrication (FFF) method with ABS and polypropylene (PP) material and the SLS method with PA2200 material. The presented research is based on a solution according to a concept consisting of four stages: firstly, make a 3D scan of the forearm; secondly, generate a 3D orthosis model; thirdly, order and manufacture the designed 3D printed orthosis; finally, wear the ordered orthosis [61,62,63]. As a result, each is designed and manufactured to meet individual needs. Concern for the possibilities of 3D printing at low- to medium-volume production is necessary.

Thanks to the research presented in this paper, it is possible to expand the information on the mechanical properties of the PA12 material to manufacture forearm orthoses using SLS and MJF technology. Samples fabricated from PA12 material using SLS and HP MJF underwent comprehensive tensile, flexural, and impact strength assessments. Strength tests considered printing the samples in five different orientations. Thanks to the knowledge of the material’s strength and deformation capacity, it is possible to design a lightweight, openwork structure for the orthosis, which reduces its weight and production and operating costs. In addition, impact testing is essential in safety assessment, as it allows us to determine how the material behaves in impact situations, which is essential when a patient falls with an orthosis on the forearm. Before printing, the PA12 material was tested using the volumetric melt flow (MVR) method for both SLS and MJF methodologies to test the feedstock’s quality before starting the 3D printing process.

In addition, SLS and MJF technologies were reviewed for producing forearm orthoses, investigating the impact of mechanical properties on production time and cost. Furthermore, the potential of PBF methods as a production tool for developing medium-sized orthopedic supplies as an alternative to plaster casts in hospital emergency departments by orthopaedists as a solution available on-site without waiting several days for a personalized product to be made was highlighted.

## 2. Materials and Methods

### 2.1. Methods of Making Test Samples

The selection of material and manufacturing technology is a crucial factor in the design of a new solution. The material choice affects wall thickness, strength, flexibility, minimum feature size, build quality, and ease of certification of the new product. Consequently, this impacts the final geometry of the orthosis, which is the subject of this study [64,65,66,67,68]. The material commonly used in PBF technology is PA12 polyamide, which is used in SLS and HP MJF technologies. In the present study, two types of polyamide 12 powder were used—PA2200 (EOS GmBH, Krailling, Germany; EOS trade name of white PA12) [69] for SLS and PA12 (HP Inc., Barcelona, Spain) [70] for HP MJF certified for biocompatibility by ISO 10993 [71]. PA2200 polyamide powder is white due to the titanium oxide additives in the polyamide 12 (PA12) material. Using titanium oxide additives in PA12 improves the mechanical properties of sintered parts, making them more suitable for a wide range of applications [69]. In SLS technology, fabrication parameters such as scan vector length, incident laser power, and energy density play a key role in the mechanical and morphological properties of the parts produced [72,73]. The color of the powder in HP MJF technology is usually grey. This is due to the use of a polymer powder, such as polyamide-12, which is heated and exposed to infrared lamps during the MJF process. The grey color of the powder is a result of the specific composition of the raw material and the manufacturing process, which distinguishes MJF from other PBF techniques, such as SLS. The use of fusing and detoxifying agents, together with a planar infrared (IR) source, contributes to the unique grey powder color used in HP MJF technology [74,75].

This study aims to characterize products manufactured by 3D printing for use in producing parts for the medtech sector. Therefore, the powder data sheet information was adopted for this purpose. In order to optimize the 3D printing process with SLS and HP MJF methods, different PA12 polyamide powders with different color shades were used due to the different sources of energy supplied (laser for SLS, heat lamp with agent factor for HP MJF). Therefore, for each PBF technology, a set of manufacturing process parameters was selected for the powder used. These data are presented in Table 1.

Samples of PA2200 powder were made in an EOS P396 machine (EOS GmBH, Krailling, Germany) [78], while PA12 powder was made in an HP MJF 5200 machine (HP Inc., Barcelona, Spain) [79]. After manufacturing, they were subjected to a finishing process of cleaning unmelted powder from them. In the case of SLS technology, the printed parts are contained in a container together with the unmelted polymer material. Unpacking them requires the container with the parts to cool down beforehand, which can take up to 12 h. Once unloaded at the unloading station, a cleaning process follows, usually carried out using compressed air or another cleaning agent. After this, the components are ready for use. In the case of HP MJF technology, the printed parts are contained in a container along with the unmelted polymer material in the build unit. The build unit is then transferred from the 3D printer to the processing station to cool down. At the processing station, a cleaning process occurs, usually carried out via compressed air or another cleaning agent analogous to the cleaning procedure in SLS technology. When the process is complete, the parts are ready for use. The parameters of the machines are shown in Table 2.

### 2.2. MVR Powder Test

To measure the thermoplastic’s melt volume flow rate (MVR), 100 g of each powder blend was prepared for testing. They were certified according to ISO 10993 [71] and were classified as intermediate products from which biocompatible components are made, as shown in Table 3.

**Figure 1:**
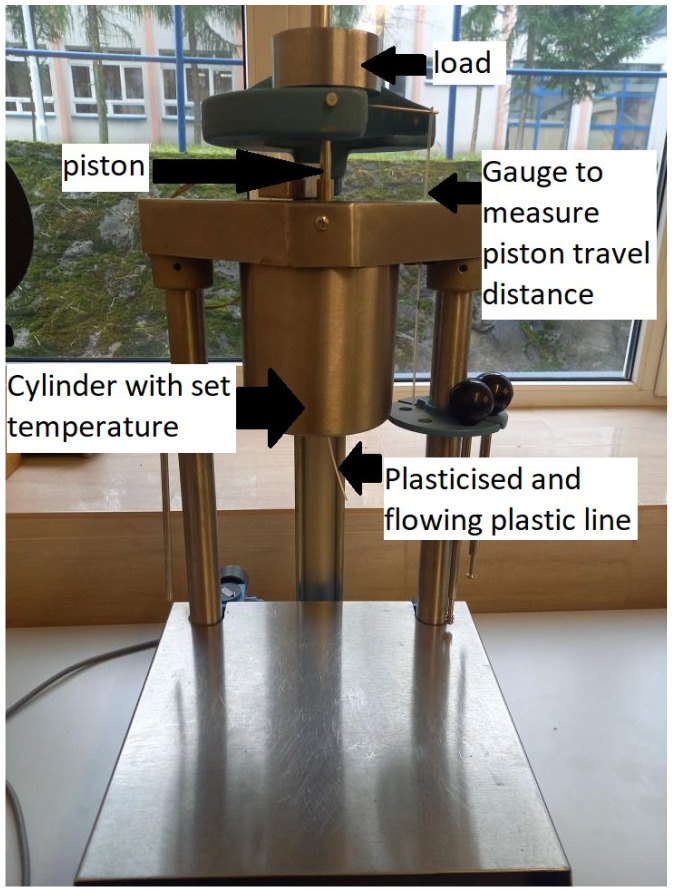
Capillary plastometer DYNISCO 4781 Kayeness.

The thermoplastics’ melt volume flow rate (MVR) was determined according to ISO 1133:2002 [80]. These parameters allow the assessment of the rheological properties of thermoplastics, which is crucial for designing and manufacturing products from these materials. MVR allows an understanding of how a material behaves during processing, including its flow ability, plasticity, and viscosity. These tests make it possible to compare different thermoplastics and select those that best meet the requirements of a given application. The MVR measurement was carried out on a capillary plastometer DYNISCO 4781 Kayeness Inc., Honey Brook, PA, USA (Figure 1), PA12 and PA2200 under the following conditions (before testing, the plastics were dried in a vacuum dryer for 4 h at 130 °C) shown in Table 4.

For this purpose, samples (mixtures of new and recycled powder for PA2200 and PA12) weighing approximately 5 g were introduced into the properly heated apparatus. A preload of 1.16 kg was applied using a balance for 30 s. After this time, the load was changed to the correct 5 kg, and measurements were taken when the length of the strand exceeded 2 cm to measure the volume after the extrusion of the polymer.

The results of MVR tests for PA2200 and PA12, produced using SLS and HP MJF technologies, can be interpreted as follows: MVR represents the volume of the molten material. A higher MVR value indicates a larger volume, while a lower MVR value indicates a smaller volume. MVR is determined by multiplying the MFR and the density of the molten sample. For PA 12, a higher MVR value indicates a greater volume of molten material, which can be important for various applications, such as injection molding or 3D printing [81,82,83,84].

The reason for conducting that test is to ensure the quality and properties of the produced plastic material and evaluate the powder’s flowability in the molten state. This is particularly important for applications in SLS and HP MJF technologies, which rely on the proper flow and adhesion of the material during the printing process [81,82,83,84].

### 2.3. Preparation and Manufacture of Specimens for Strength Tests

In order to obtain the characteristics of the PA12 polyamide material group, test samples oriented in 5 different directions were examined and compared: flat (X), edge (Y) and vertical (Z), tilted by 45° in the XZ axis, and tilted by 45° in the YZ axis in to assess the anisotropy of 3D printed materials:

- Based on ISO 527 [85], tensile test specimens were designed on an Instron 5985 universal quasi-static testing machine (Instron Corp, Norwood, MA, USA) with a maximum load of 250 kN;
- Based on ISO 178 [86], flexural test specimens were designed on an Instron 5985 universal quasi-static testing machine with a full load of 250 kN;
- Based on ISO 179 [87], impact test specimens were made on an Instron CEAST 9050 machine (Instron Corp, Norwood, MA, USA) with a hammer energy range of 0.5–50 J.

**Figure 2:**
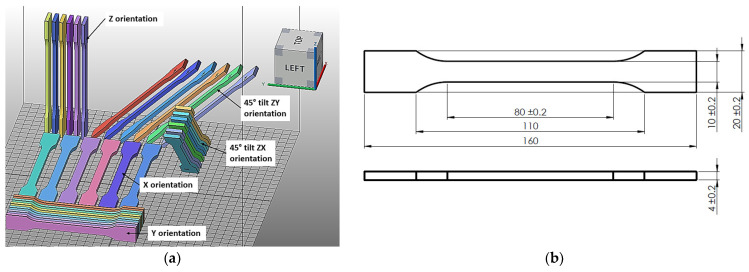
Preparation of specimens to be made from polyamide PA12 material on EOS P396 and HP MJF 5200 machines. Arrangement of ISO 527 tensile specimens in the working chamber of the machine (**a**). Tensile test specimen dimensions in accordance with ISO 527 (**b**).

**Figure 3:**
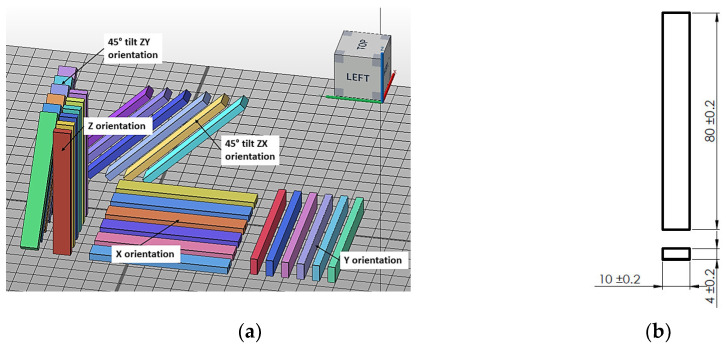
Preparation of specimens to be made from polyamide PA12 material on EOS P396 and HP MJF 5200 machines. Arrangement of ISO 178 flexure specimens in the working chamber of the machine (**a**). Flexural test specimen dimensions in accordance with ISO 178 (**b**).

**Figure 4:**
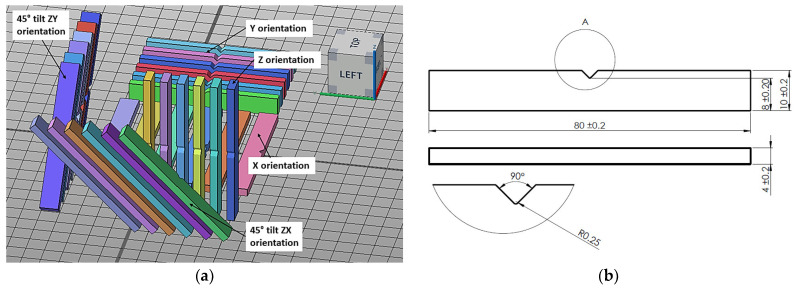
Preparation of specimens to be made from polyamide PA12 material on EOS P396 and HP MJF 5200 machines. Arrangement of ISO 179 impact specimens in the working chamber of the machine (**a**). Impact test specimen dimensions in accordance with ISO 179 (**b**).

**Figure 5:**
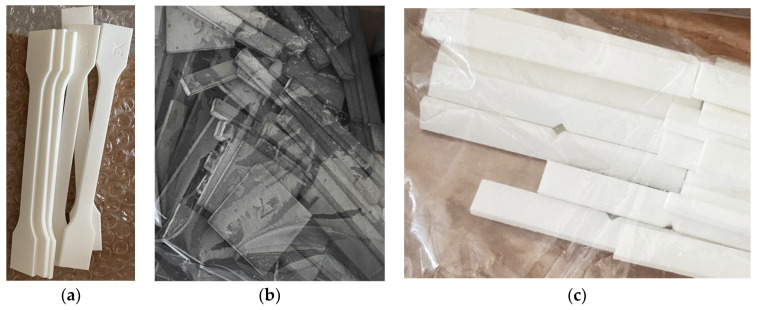
ISO 527 tensile test specimens (**a**); ISO 178 flexural test specimens (**b**); ISO 179 impact test specimens (**c**); white color—SLS technology specimens; grey color—HP MJF technology specimens.

Table 5 and Figure 2, Figure 3, Figure 4 and Figure 5 show the designed test samples.

### 2.4. ANOVA Analysis for Mechanical Tests

In the statistical analyses, the confidence interval for the mean was calculated for a confidence level of 95%. In the analyses of variance carried out, the level of significance was assumed to be ∝ = 0.01. This was due to the performance of multiple statistical tests, which increased the probability of a type I error. The post hoc tests conducted after the analysis of variance were Tukey–Kramer HSD tests. All statistical analyses were performed in JMP 12 (SAS Institute Inc.: Cary, NC, USA) software [88].

### 2.5. Preparation of the Analysis of the Manufacturing Process—Analysis in a CAM Environment—Case Study of Forearm Orthoses

In order to analyze the manufacturing process using PBF technology, components were selected for SLS and HP MJF technology. The forearm orthosis manufactured using 3D printing technology consists of 3 separate components—the upper component of the orthosis frame—designated number 001; the lower component of the orthosis frame—designated number 002; and the thumb grip—designated number 003. All the above 3 components make up an integrated forearm orthosis assembly. These are shown in Table 6 and Table 7. The forearm orthoses were designed in SolidWorks 2022 [89]. After designing them using the writing to STL function, the software prepared and read files to prepare the 3D printing process for PBF technology. To analyze the manufacturing process in a computer-aided manufacturing (CAM) environment in Materialise Magics 25 [90] and Autodesk Netfabb 2023 [91] software, performance analyses were carried out for medical devices from Mediprintic sp. z o.o (Mielec, Poland).

Because of the stacking of 3D models in the virtual chamber of the 3D printer, it uses the following methods [92]:

- Manual component placement carried out by an experienced technologist.
- 3D packing scanline—during the packing process, the parts rotate as necessary and according to user-defined settings to find the best orientation for denser packing. Once packing begins, the calculations continue until they are completed.
- 3D packing Monte Carlo (the Monte Carlo method is a technique used for the mathematical modeling of processes that are too complex, such as the calculation of integrals or chains of statistical processes. It involves the use of random sampling of quantities that characterize a process according to a known distribution [93])—during the packing process, if the settings allow, the parts are rotated at the beginning but not afterward. The process is similar to random shuffling, where parts move into gaps as they find them. Monte Carlo method packing aims to move the parts as low as possible in the compilation space to minimize platform height.
- 3D packing gravity—allows parts to self-settle in the production room under simulated gravity.
- 3D packing size sorting—is a combination of Monte Carlo and Scanline, focusing on the requirement first to pack large parts and stack them towards the center of the platform. Then, in that order, medium and small parts fill gaps and achieve a high overall packing density in the process.

Using the available tools in the CAM environment, the authors arranged the maximum number of orthoses that could be accommodated in the working chambers of the EOS and HP machines. Parameters such as packing density and the total volume occupied by the components in the working chamber, which are key parameters affecting the manufacturing process, were measured using the analysis tools available in the software. Then, using the dedicated software, analyses were carried out on the duration of the manufacturing process.

An analysis of the production possibilities for the best alignment of the 3D models in a 45° tilt in terms of the mechanical properties obtained and for the highest packing in light of the density of the filling of the working chamber was presented to compare the production possibilities. The analysis was based on data on forearm injuries in the Hospital Emergency Department of the Independent Public Clinical Hospital No. 1 of the Pomeranian Medical University in Szczecin. There were 263 forearm injuries in July and 214 in December, indicating a demand for 477 forearm orthoses using 3D printing technology during these two months [94].

The authors conducted a study comparing the production performance of the EOS P396 and HP MJF 5200 machines. They considered the following parameters in the analysis:

- Maximum number of parts to be stacked in the working chamber;
- Density of the chamber;
- Total volume of elements in the working chamber;
- Manufacturing time, cooling time, process start-up time;
- Price per material used.

However, there is a specific disclaimer: SLS and MJF systems have different performance levels in powder use. The HP MJF 3D printer requires parts to be widely spaced apart to reduce the potential for heat build-up. The recommended packing density is limited to around 8–10%, meaning that as much material goes into the printed parts, it will need to be discarded. This means that HP MJF systems produce a lot of non-recyclable powder, which increases ongoing printing costs. With SLS technology, the recommended packing density is limited to 15%. For both of these large systems, the energy required to run 3D prints means that starting with just a few small parts is inefficient, highlighting that only manufacturers with predictable, high-volume 3D printing needs [95].

It is worth noting that the components shown in the benchmark were designed per the Design for Additive Manufacturing methodology, which focuses on consolidating multiple components into a single functional object and using lattice structures [96].

## 3. Results and Discussion

### 3.1. MVR Results

During the measurement, the time taken for the appropriate section of plastic to flow out of the capillary was determined. Based on the results obtained, the MVR was calculated according to the following formula, and the results obtained are summarised in Table 8.

(1) $$MVR\left(T,m\right)=\frac{A600l}{t}$$

*T*—test temperature [°C]
*m*—load [kg]
*A*—arithmetic mean of the cross-sectional areas of the cylinder and piston head [cm^2^]
*T*—measurement time [s]
*l*—piston displacement distance [cm]

**Table 8:** Test results for the volumetric melt flow rate MVR [cm^3^/10 min].

Material	185 °C	190 °C
PA12	-	7.521 ± 0.553
PA2200	3.543 ± 0.946	11.385 ± 0.883

Based on the material data, MVR measurements were carried out in the upper melting temperature range of the plastics, i.e., 185 °C for PA2200 and 190 °C for PA12. However, in the case of PA2200, inhomogeneous plasticization of the plastic was observed, which resulted in a considerable variation in results. Therefore, for PA2200, MVR measurements were also carried out at 190 °C, resulting in better plastic flow and the possibility of accurately determining the tested parameter. The MVR values obtained in the MVR test presented in Table 8 are in accordance with the accepted values in ISO 1133-1:2011 and other tests in the range of 0–50 cm^3^/10 min [97,98]. Therefore, obtained values are measurable parameters to refer to as appropriate parameters for characterizing the polyamide powder blend for SLS and HP MJF technologies in forearm orthosis application.

It is essential to consider the specific requirements of the application and the desired material properties when interpreting the results of MVR tests for PA2200 and PA12 produced using SLS or HP MJF technologies. In conclusion, the MVR results indicate that powdered plastic blends according to the proportions for variants 1 and 2 can produce biocompatible components using 3D printing technology.

### 3.2. Tensile Test

The results of an ANOVA for repeated measures investigating the effect of the manufacturing method on the values of a given tensile test parameter are shown in Table 9. The same table also shows the results of analyses of variance investigating the effect of model orientation—carried out separately for SLS and MJF specimens—on the tensile parameters. Assuming a significance level of ∝ = 0.01, the ultimate tensile strength and tensile modulus of the SLS and MJF fabricated samples are statistically equal. However, the fabrication method has a statistically significant effect on the tensile elongation at break ε*_t_*. This is mainly due to the significantly higher ε*_t_* values of the SLS specimens in X and Y orientations.

The model’s orientation in printer space statistically affects all tested strength parameters—for both SLS and MJF samples. As for tensile strength, the Tukey test showed that the strength of SLS samples printed in XZ orientation is higher than in X, Y, and YZ. The difference in ultimate tensile strength between XZ (with the highest σ*_t_*) and YZ samples (with the lowest value of σ*_t_*) was 4.1 MPa. In the case of MJF samples, the statistically significant effect of orientation on ultimate tensile strength was due to the lower strength of samples printed in the Y direction relative to all other samples.

**Figure 6:**
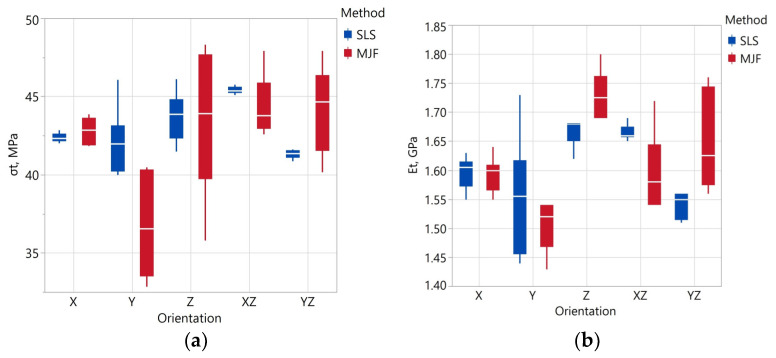
Ultimate tensile strength $\sigma_{t}$ (**a**) and tensile modulus $E_{t}$ (**b**) depending on fabrication method and specimen orientation.

**Figure 7:**
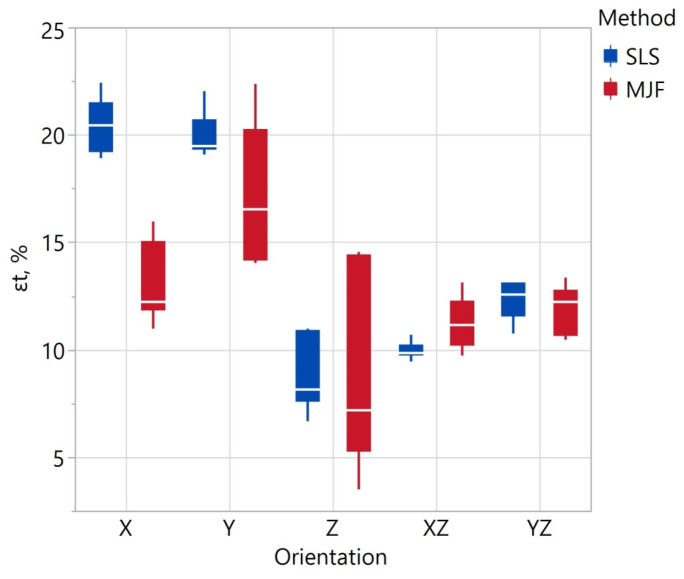
Tensile elongation $\varepsilon_{t}$ depending on fabrication method and specimen orientation.

The results of the strength parameters of the specimens obtained from the tensile tests are shown in Table 10 and Figure 6 and Figure 7. The results shown in Figure 6 clarify that the ultimate tensile strength and tensile modulus values in the same orientation as the values obtained for the SLS and MJF specimens are not significantly different. The average σ*_t_* value of all SLS specimens was 43.05 ± 0.71 MPa, and that of the MJF specimens was 42.31 ± 1.52 MPa. Therefore, the average values differ by about 1.8%. The smallest value of σ_t_ = 41.32 ± 0.34 of the SLS samples was recorded for the YZ orientation, and the MJF σ_t_ = 36.76 ± 3.52 at the Y orientation.

Similar tensile strength tests of SLS samples, but printed only in three orientations (X, Y, Z) on EOS devices, were carried out by the authors of publications [47,99,100,101], among others. The tensile strength values we obtained of 41–45 MPa are similar to those presented by [47,100,101]. Lammens [99] obtained slightly higher σt values (45.0–49.4 MPa), while Calignano [51] obtained significantly lower σt values (34.8–38.3 MPa). Analogous strength tests in three orientations for the MJF method are presented in the literature [47,100,102]. Tensile strength values of 36.5–44.5 MPa, similar to those obtained by us, were obtained by Mehdipour [47]. Calignano [51] received noticeably lower σt values (34–38 MPa), while in [101] and Morales [49] received the highest σ_t_ values above 47 MPa. Due to the layered structure of printed models, the topic of anisotropy is raised when analyzing tensile strength. In the case of tensile strength, it is expected that models for which the direction of force is consistent with the direction of model building are less intense [103]. However, the anisotropy of the properties depends mainly on the bonding energy. The bonds between successive layers are smaller at low bonding energy, and the model has more significant anisotropy. Increasing the bonding energy makes the model properties more isotropic [76]. In our study, as in the publications [47,100,103], the lowest strength was not associated with samples printed in Z orientation, i.e., in the direction of tensile force.

Depending on the orientation, the tensile modulus value E_t_ of the SLS samples varied in the range of 1.54–1.67 GPa and that of the MJF samples in the range of 1.51–1.73 GPa. The smallest value of E_t_ for SLS was recorded in the YZ orientation and for MJF samples in the Y orientation. Similar E_t_ values of SLS and MJF samples were reported by the authors of [47,48], among others. The E_t_ values reported by Calignano [51] are similar for SLS samples but slightly lower (1.2–1.4 MPa) for MJF samples.

The orientation of the model had the most significant effect on tensile elongation. The largest ε_t_ values were obtained for the X and Y orientations. SLS specimens exhibited more excellent elongation in these orientations in the order of 20%. The anisotropy of the strength properties of the models resulting from the layered structure is, therefore, mainly manifested in the tensile elongation. This is also confirmed by other test results [47,99,100,101,102].

Compared to the MJF specimens in a given orientation, the SLS specimens showed less variation in the parameters obtained from the tensile test. This indicates that the properties of the SLS samples are more repeatable (assuming 3D printing in the same orientation). The coefficients of variation of ultimate tensile strength and tensile elongation calculated for all samples in the case of SLS are also smaller. This means that the SLS samples had an overall greater homogeneity of the parameters, as mentioned earlier. The more significant coefficient of variation of the SLS specimens in the case of tensile elongation is due to the significantly higher elongation of the specimens 3D printed in X and Y orientation.

### 3.3. Flexural Test

**Figure 8:**
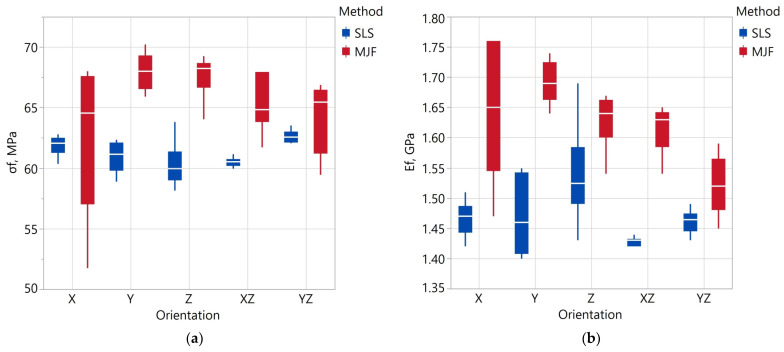
Flexural strength $\sigma_{f}$ (**a**) and flexural modulus $E_{f}$ (**b**) depending on fabrication method and specimen orientation.

**Figure 9:**
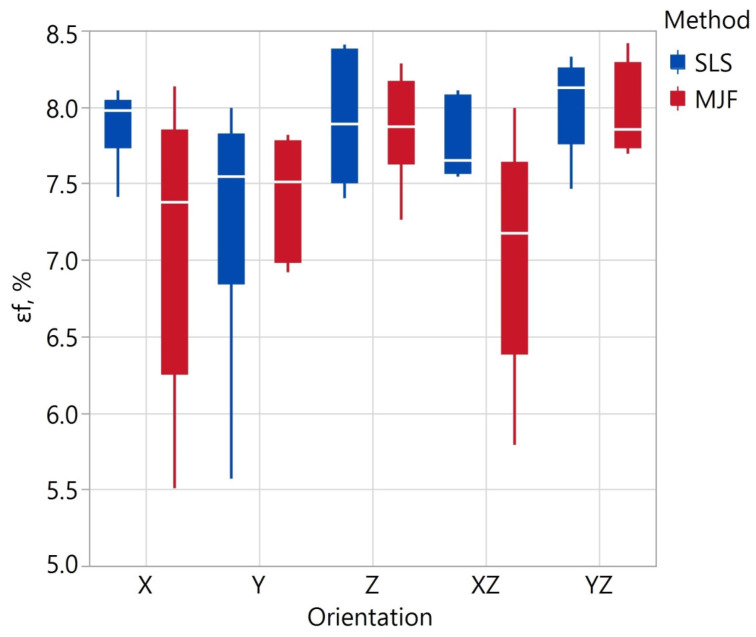
Flexural elongation $\varepsilon_{f}$ depending on fabrication method and specimen orientation.

The results of the strength parameters of the specimens obtained in the flexural tests are presented in Table 11 and Figure 8 and Figure 9. Table 12 shows the results of the ANOVA analysis of variance for repeated measures investigating the effect of the manufacturing method on the values of a given parameter from the bending test. The same table also shows the results of variance analyses investigating the effect of model orientation—carried out separately for SLS and MJF specimens—on the strength parameters. Assuming a significance level of ∝ = 0.01, the chosen manufacturing method had a statistically significant effect on flexural strength and flexural modulus.

For SLS specimens, model orientation had a statistically significant effect on flexural strength. The statistical significance is due to the differences between the values obtained for the YZ, XZ, and Z orientations. These differences are relatively small (approximately 3%), as seen in Figure 9. For MJF specimens, the model’s orientation had a statistically significant effect on flexural modulus.

The specimens made by the MJF method showed higher flexural strength by 4.7%. The average σ_f_ value of all SLS specimens was 61.25 ± 0.53 MPa, and that of MJF specimens was 65.73 ± 1.45 MPa. The smallest value of σ_y_ = 60.30 ± 2.01 of the SLS specimens was recorded for the Z orientation, and for the MJF specimens, σ_y_ = 62.77 ± 8.15 at the X orientation. The observed orientation effect on σ_y_ is consistent with the results presented in [101]. In [102], MJF Z specimens had the highest flexural strength. This was due to the lowest porosity of these samples. The values of σy reported in [101] are smaller than in this study and vary in the 46.3–57.7 MPa range. On the other hand, the values reported in [102] have more significant variation (50–70 MPa).

A higher flexural modulus also characterized the MJF specimens. Depending on the orientation, the tensile modulus value E_f_ of the SLS specimens varied in the range of 1.43–1.54 GPa, and that of the MJF specimens in the range of 1.52–1.69 GPa. The smallest value of E_f_ for SLS was recorded in the Z orientation and for MJF samples, in the X orientation. The mean values of flexural elongation depending on the orientation for the SLS samples varied in the range of 7.3–8.0%, and for the MJF samples in in the range of 7.0–8.0%. The observed values for the MJF samples are similar to those presented in the publication [102]. The authors of [101] reported lower E_f_ values in the range of 0.87–1.07 GPa.

Compared to the MJF specimens in a given orientation, the SLS specimens showed less variation in the parameters obtained from the flexural test. This shows that the properties of the SLS samples are more repeatable (assuming printing in the same orientation). The coefficients of variation calculated for all samples in the case of SLS are also smaller. This means the SLS samples had an overall greater homogeneity of the analyzed parameters related to the flexural test.

### 3.4. Impact Test

**Figure 10:**
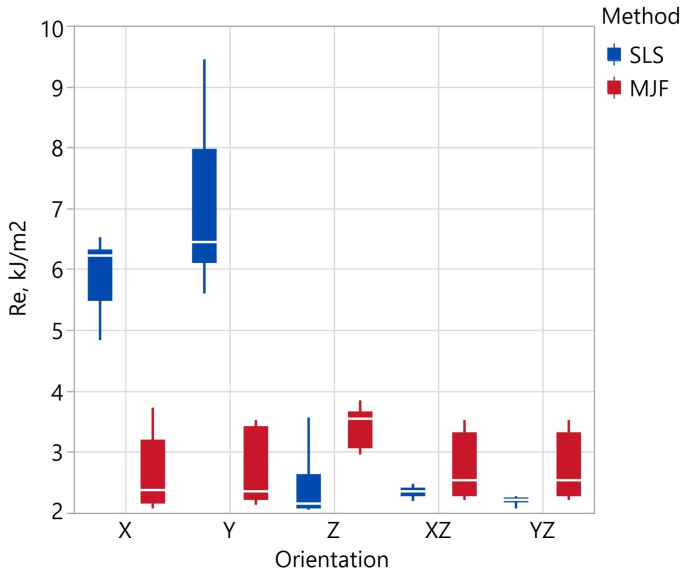
Impact strength *R_e_* depending on fabrication method and specimen orientation.

The impact test results are shown in Table 13 and Figure 10. The analysis of variance showed that the printing method has a statistically significant effect on impact strength (*p*-value = 0.003). The orientation of the print had a statistically significant effect on the R_e_ value only for SLS samples. For SLS samples printed in the X and Y orientation, impact strengths of approximately 6 and 7 kJ/m^2^ were determined, respectively. These values are approximately 1.5–3 times higher than the others. As noted earlier, the SLS samples in the X and Y orientation also had significantly higher tensile elongation, which is related to the anisotropy of the SLS samples. The smallest value of R_e_ = 2.21 ± 0.07 kJ/m^2^ of the SLS samples was recorded for the YZ orientation and for the MJF R_e_ = 2.63 ± 0.65 kJ/m^2^ at the X orientation. A minor mean variation in impact strength (calculated as the average of the CVs from the different orientations) was observed for the SLS samples. This means that SLS samples exhibit more excellent uniformity in the R_e_ parameter when printed in the same orientation. The higher coefficient of variation of all SLS samples is due to the significantly higher impact strength of samples printed in the X and Y orientations. Publication [104] also studies the impact strength of SLS and MJF samples from PA12 in X and Z orientation. Most samples had similar impact strengths of 2.1–2.7 kJ/m^2^. The exception was the X-oriented MJF sample, for which R_e_ was about 5 kJ/m^2^.

### 3.5. Result of Capacity Analysis in 3D Printers—A Case Study of Forearm Orthoses

In order to optimize the positioning of parts in the working chambers of the EOS P396 and HP MJF 5200 machines, 3D packing size and manual sorting methods were used to position the medical components—forearm orthoses—best. The component stacking process was carried out in Autodesk Netfabb 2023 and Materialise Magics 25, which is integrated by default with the 3D printers offered by EOS and HP. When the Autodesk Netfabb 2023 and Materialise Magics 25 [95,105] software launches, a list of available 3D printers opens, with process parameters and configured build platforms. Accordingly, the user focuses on arranging the parts in the working chamber using packaging tools like 3D packing Monte Carlo.

**Figure 11:**
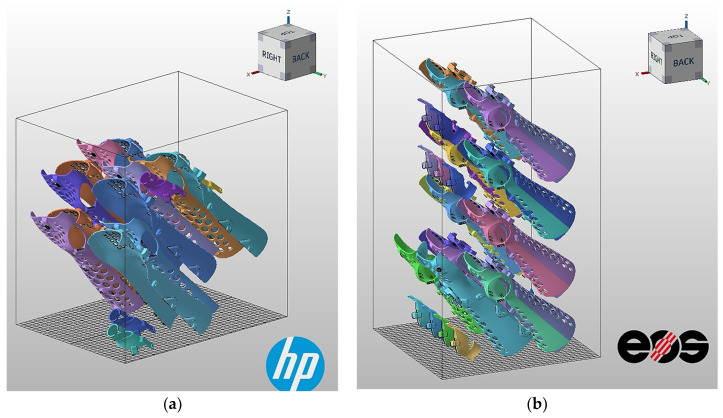
Case study of component alignment based on the best strength properties in the working chamber of a 3D printer for forearm orthoses; medical component alignment in HP MJF 5200 (**a**); alignment in EOS P396 (**b**).

**Figure 12:**
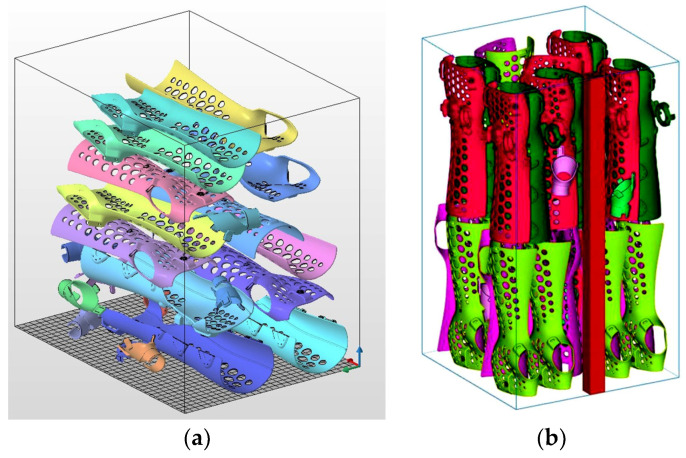
Case study of component alignment based on the maximum elements in the working chamber of a 3D printer for forearm orthoses; medical component alignment in HP MJF 5200 (**a**); alignment in EOS P396 (**b**).

Two cases were investigated in the analysis, the first for the best mechanical parameters obtained from the tensile test (XZ tilted 45 degrees)—optimized for mechanical properties; the second for the maximum filling of the working chamber with forearm orthoses—optimized for economic efficiency. The SLS technology on the EOS P396 machine produced 9 sets of M-size orthoses sets (three components) for the first option and 15 sets for the second option. In comparison, the HP MultiJet Fusion technology on the HP MJF 5200 produced 7 sets of S-size orthoses for the first option and 11 sets for the second option. The difference between the volumes of the models between sizes M and S was 7.9%. The results of the production capacity for each machine are shown in Table 14 and Figure 11 and Figure 12.

In the case of the HP MJF technology, the cooling down process is carried out in a separate machine, so the total time associated with the 3D printing process in the HP MJF 5200 is 12.35 h, and the remaining 11 h of the process associated with cooling down and unpacking the working chamber is 11 h. In the case of SLS technology, the entire process, from 3D printing to the cooling down process, takes place in one machine, which ultimately takes 34.88 h. Significantly, the production time depends on the height of the parts in the working chamber. For the EOS, the height of the arranged elements was 570 mm; for the HP, it was 380 mm, so the production times are the same for both variants (1 and 2).

Considering the weekly production capacity for SLS, it is possible to run three production processes, equivalent to producing 27/45 sets (variant 1/variant 2) of orthoses. At the same time, for HP MJF, it is possible to start five production processes, which make 35/55 (variant 1/variant 2) sets of orthoses. Operating assumptions have been made for single-shift operation and a working week of Monday to Friday (therefore, the JOB 3 chamber will be unpacked on Monday of the following week), as shown in Table 15 and Table 16.

A capacity analysis was carried out on the machine capacity side for variant (option) 1 and variant (option) 2 is shown in Table 17.

From the comparison of production capacities made, the following conclusions can be reached:

- More parts can be made in one production cycle on the EOS P396 machine than on the HP MJF 5200;
- Total production costs are lower for HP MJF technology than SLS;
- The HP MJF 5200 system has a higher weekly production capacity than the EOS P396 by 30% for option 1 and 18.2% for option 2;
- Manufacturing components with high mechanical properties is 13% more expensive for SLS and 4% more expensive for HP MJF than the variant associated with economic efficiency;
- The filling density of the parts in the working chambers is lower than the recommended packing density of the parts, as it is below 10% of the parts will not warp due to their packing density in the working chamber;
- From the side of the delivery time of the finished orthosis, the most efficient is the HP MJF 5200 system in the economical variant;
- For the case study presented, total production costs are 32.6% lower for option 1 and 26.6% lower for option 2 between HP MJF and SLS;
- From the point of view of the powder used and its cost, it is more cost-effective to produce on the HP MJF 5200 system.

Within the context of investigating the manufacturability of a 3D-printed forearm orthosis, similar studies have been conducted using FFF technology for ABS, PLA, HIPS, and nylon materials in which criteria related to manufacturing accuracy with regard to manufacturing time and cost were investigated. The results of the obtained studies indicated a significant influence of mechanical parameters on the geometry of the orthosis depending on the positioning of the 3D model in the working chamber of the 3D printer. However, it is essential to consider the high anisotropy of the mechanical properties of 3D printing with FFF technology [35,55,107].

### 3.6. Result of Defects Analysis in 3D Printing—Case Study of Forearm Orthoses

Based on the manufacturing processes performed with SLS and HP MJF technology, potential problems for the production of forearm orthoses were investigated, such as [108]:

1. Surface defects. Unfavorable surface properties of the printed parts can result from the sedimentation process of the material or imperfections in the 3D printing technology itself. This problem can be solved by optimizing the positioning of the workpiece in the 3D printer’s work chamber space. In order to obtain the best surface quality for SLS technology, the relevant surfaces of the orthosis should be inverted with the *Z*-axis manufacturing direction. As well as for HP MJF technology, the relevant surfaces of the orthosis should be oriented towards the *Z*-axis manufacturing direction.
2. Geometric distortion. Geometric distortion may occur as a result of internal stresses. In the present case, the filling density of the working chamber is below the critical values for optimal filling of the chamber, so there is no reason for this phenomenon to occur.
3. Cracks. These can occur as a result of material inhomogeneity, inadequate cooling, or excessive stress. For this purpose, it is essential to check the machine settings and its calibration—especially the laser source in the SLS and the heating lamps in the HP MJF—before starting the 3D printing process.
4. Material quality problems. Material defects, such as impurities or inhomogeneities, can lead to defects in the prints. To avoid this, quality control of the powder mixture should be carried out before production starts.

**Figure 13:**
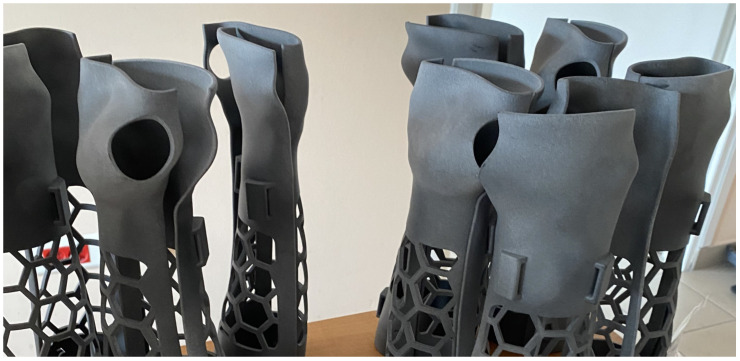
Forearm orthoses production example made in HP MJF technology.

**Figure 14:**
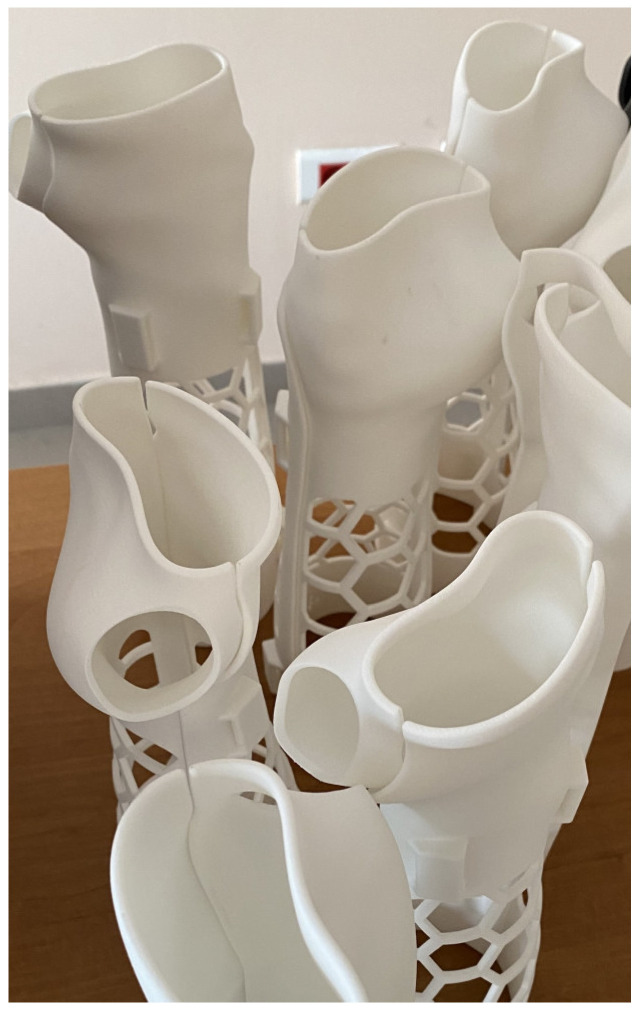
Forearm orthoses production example made in SLS technology.

Furthermore, these defects can occur for various reasons, including printing parameters, material quality, equipment maintenance, or even the 3D model design. Therefore, the HP MJF 5200 (orthoses examples showed in Figure 13) and EOS P396 (orthoses examples showed in Figure 14) must take care of the equipment’s annual servicing and calibration and carry out quality control for the powder mixes. An essential factor is to control the quality of the STL file from which the actual product is created and to control the nesting density of the working chamber so that its maximum density does not exceed 10%. The element packing analysis performed indicates a direction in establishing a manufacturing criterion for forearm orthoses. In the case of an analysis based on maximizing the critical mechanical property parameters, a low packing density was obtained, similar to the case of maximizing the elements in the working chamber of the 3D printer. Therefore, in the case of the production variants presented in the form of option 1 and option 2 from the point of view of the appearance of potential production defects is not significant due to the low working chamber density in the range of 1.8% to 3.7%. Therefore, an essential factor affecting capacity is the delivery of a certain number of orthoses that satisfy the end customer’s demand.

## 4. Conclusions

The purpose of this research work was to investigate the mechanical properties of polyamide PA12 as a 3D printed material using Selective Laser Sintering (SLS) and HP MultiJet Fusion (HP MJF) technologies to design and manufacture forearm orthoses. The study evaluated the flowability of the materials used and compared the mechanical properties of PA12 with each other using tensile, flexural, and impact tests in five different manufacturing orientations. In addition, analyses were conducted on the manufacturing process—a case study of forearm orthoses.

The MVR analysis carried out allows the quality of the PA12 and PA2200 polyamide blend used to determine the properties of the HP MJF and SLS technologies. Moreover, the measurement results obtained from the MVR test can be used to assess the repeatability of production for an ordered batch of orthoses in order to determine the quality of the powder mixtures for each manufacturing process in the 3D printer. The results obtained from the ISO 527 tensile test could be used to compare the mechanical properties of the orthoses between different manufacturing processes on the same 3D printer. In practice, two ISO 527 tensile specimens, for example, can be added to each working chamber, and then a tensile test can be carried out to compare the mechanical properties with the results obtained in the tests to evaluate the mechanical performance of the manufactured forearm orthoses.

The ISO 527 tensile test, ISO 179 bending test, and ISO 178 impact test provided important information on the mechanical properties of the polyamide powder blends used for the selected technologies. The data collected are important from the perspective of the design of a 3D-printed forearm orthosis from the point of view of its strength properties and evaluation of the safety of the final product. From the point of view of the 3D-printed forearm orthosis user, what is important is orthosis stiffness, susceptibility to flexure, and resistance to collisions such as a fall or accidental impact by another person.

Regarding the tests carried out and the analysis of the results, the following conclusions are presented:

1. The PA2200 and PA12 powder blends used are suitable for producing biocompatible components, which is confirmed by certificates from the manufacturers EOS and HP MJF. The values obtained in the MVR test for PA12 and PA2200 powders were about 7.5 and 11.4 cm^3^/10 min, respectively.
2. SLS samples had an average 1.8% higher tensile strength than MJF samples. MJF samples, on the other hand, had a 4.7% higher flexural strength. Therefore, from the point of view of the forearm orthosis manufacturer, it is appropriate to analyze the production costs to the expected mechanical properties of the final product.
3. SLS specimens in a given orientation have less variability in mechanical properties than HP MJF specimens. Therefore, more repeatable mechanical parameters can be predicted by producing in the specified orientation components using SLS technology relative to HP MJF technology. On the other hand, MJF samples were more isotropic—their mechanical properties were less dependent on orientation.
4. For the presented case study, it is possible to select tensile samples from the Y orientation for each 3D printing technology in which the smallest mechanical values were obtained as a reference for assessing the reproducibility of orthoses made by several manufacturing processes.
5. This is important information from the point of view of selecting the wall thickness of the orthosis in relation to its strength, which ultimately translates into the weight of the plaster. In the case in question, the total weight of the orthosis is about 180 g for size M. At the same time, the classic white cast weighs about 1 kg, so we have a 5× reduction in weight while maintaining the rigidity that stabilizes the forearm in the correct position.
6. The orientation of the 3D model alignment in the working chamber of the 3D printer significantly affects strength parameters such as tensile strength, flexural strength, and impact strength. In order to verify the necessary mechanical parameters of the orthosis, strength analysis should be carried out based on the data obtained [109,110], e.g., in Ansys 2023 R2 software, in order to investigate the occurring stresses in relation to the biomechanics of the forearm. The best solution is to tilt the 3D models in the working chamber of the machine. The process’s economics should also be considered when analyzing the total cost of manufacturing forearm orthoses.
7. The results obtained from the capacity analysis indicate to the 3D-printed forearm orthosis manufacturer the direction in which it is important to focus on the development of the product from the point of view of its mechanical properties and production capabilities.
8. Total production costs for HP MJF technology are 11.5% lower than for SLS technology. Manufacturing components with high mechanical properties is 13% more expensive for SLS and 4% more expensive for HP MJF than the variant associated with economic efficiency. From the side of the delivery time of the finished orthosis, the most efficient is the HP MJF 5200 system in the economical variant.
9. The EOS P396 system allows more orthoses to be made simultaneously in a single process than the HP MJF 5200. However, during the course of one manufacturing cycle on the EOS machine, it is possible to perform two manufacturing cycles on the HP machine, resulting in a more significant number of forearm orthoses produced. To sum up, the HP MJF 5200 system has a higher weekly production capacity than the EOS P396 by 30% for the optimized mechanical properties variant as well as 18.2% for the optimized economical efficiency variant.
10. The application of 3D printing technology for small batch, prototyped production is applicable for medical devices as a solution for their immediate production. The proposed PBF technologies can replace conventional methods such as injection molding or milling and produce a series of up to several dozen sets in a weekly cycle.
11. Adopting a production-based solution using PBF technology reduces production start-up costs due to the lack of need to design and manufacture industrial molds needed for injection molding, among other things. Based on the presented case study, it is possible to schedule in advance the production of orthoses that will meet the volume requirements for a hospital placing an order for a medium-sized batch of forearm orthoses.
12. The choice of the manufacturing method for forearm orthoses focuses primarily on optimum mechanical properties, production capacity, and manufacturing costs, as the materials used, PA12 and PA2200, have similar mechanical properties.
13. It is possible to interchangeably use both the EOS P396 and the HP MJF 5200 in order to meet market needs and to be able to choose the shade of the orthosis in white or grey due to customer’s needs of ordered orthosis sets.
14. Potential additive manufacturing defects such as surface defects, geometric distortion, cracks, and material quality problems that a medical device manufacturer using 3D printing technology should pay attention to are diagnosed. One solution is to control the filling density of the working chamber to a maximum of 10%.
15. The results of the studies obtained allowed for the implementation of the presented SLS and HP MJF technologies as certified methods of production of forearm orthoses and for registration as a medical device at the Office for Registration of Medicinal Products, Medical Devices, and Biocidal Products and EUDAMED.
16. The applied materials, PA12 and PA2200, are used in producing forearm orthoses at Mediprintic sp. z o.o. due to their mechanical properties and production capability.

The research and development work was integrated into existing trends based on zero waste production and Industry 4.0 to achieve sustainable development goals. The strength test results were applied to the ORT Light forearm orthosis design manufactured by Mediprintic sp. z o.o.

The strength tests carried out for five different orientations can be used to develop a material model for static strength analyses [109,110]. Further development directions may be directed at studying the impact of the mechanical properties of 3D-printed forearm orthoses subjected to finishing treatments such as dyeing, mechanical polishing, and chemical smoothing. Similarly, research and development efforts are needed to optimize the design of forearm orthoses to correct production capacity and performance.

## Figures and Tables

**Table 1:** Parameters of the powders used to produce the samples [76,77].

Powder’s Name	PA2200	PA 12
Average grain-particle size	56 µm	60 µm
Bulk density	0.45 g/cm^3^	0.425 g/cm^3^
Powder melting point	176 °C	187 °C
Density of parts	0.93 g/cm^3^	1.01 g/cm^3^

**Table 2:** Parameters of the equipment on which the samples were made [78,79].

Manufacturing Method	HP MultiJet Fusion	Selective Laser Sintering
3D Printer	HP MJF 5200	EOS P396
Building volume	380 mm × 284 mm × 380 mm	340 mm × 340 mm × 600 mm
Building speed	Up to 0.014 m/s	Up to 6 m/s
Layer thickness	0.08 mm	0.12 mm
Sintering energy source	heating lamps	laser
Average power consumption	12 kW	2.4 kW
Power supply	380–415 V, 50 A max	400 V/32 A

**Table 3:** Powder mixture used as a mixture of new powder and recycled powder [73,74].

Material	PA12	PA2200
Fresh powder	20%	50%
Recycled powder	80%	50%

**Table 4:** MVR measurement parameters.

Parameter	PA12	PA2200
Preload [kg]	1.16	1.16
Basic load [kg]	5	5
Plasticisation time [s]	300	300
Plasticisation temperature [°C]	190	185, 190

**Table 5:** Summary of completed strength test specimens: tensile test, flexural test, impact test.

Technology	Material	Type of Sample and Their Orientation	Number of Samples
SLS	PA2200	tensile test, flexure test, impact test samples in X, Y, Z, tilted 45° XZ (XZ), tilted 45° YZ (YZ)	6 samples in each orientation for ISO 527 tensile test, ISO 178 flexure test, ISO 179 impact test
HP MJF	PA12	tensile test, flexure test, impact test samples in X, Y, Z, tilted 45° XZ (XZ), tilted 45° YZ (YZ)	6 samples in each orientation for ISO 527 tensile test, ISO 178 flexure test, ISO 179 impact test

**Table 6:** Part benchmark for SLS technology and PA2200 material.

Part Name	M_RIGHT_001	M_RIGHT_002	M_RIGHT_003
Part picture	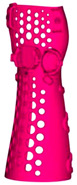	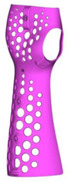	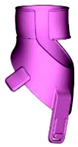
Volume	83.14 cm^3^	57.68 cm^3^	10.07 cm^3^
Weight	~77.3 g	~53.6 g	~9.4 g

**Table 7:** Part benchmark for HP MJF technology and PA12 material.

Part Name	S_RIGHT_001	S_RIGHT_002	S_RIGHT_003
Part picture	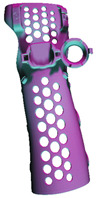	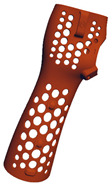	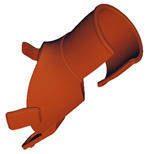
Volume	76.60 cm^3^	49.17 cm^3^	8.04 cm^3^
Weight	~77.4 g	~46.7 g	~8.1 g

**Table 9:** Results (*p*-value) of ANOVA for repeated measures investigating the effect of manufacturing method and model orientation on the parameter values obtained in the tensile test.

Effect	$\sigma_{t}$	$E_{t}$	$\varepsilon_{t}$
Method	0.295	0.606	0.009
Orientation—SLS	<0.001	<0.001	<0.001
Orientation—MJF	0.001	<0.001	<0.001

**Table 10:** Descriptive statistic of ultimate tensile strength $\sigma_{t}$, tensile modulus $E_{t}$, and tensile elongation $\varepsilon_{t}$ of SLS and MJF specimens.

Method	Orientation	$\sigma_{t}$, MPa			$E_{t}$, GPa			$\varepsilon_{t}$, %		
		Mean	Std	CV	Mean	Std	CV	Mean	Std	CV
SLS	X	42.4	0.29	0.69	1.6	0.03	1.76	20.49	1.27	6.21
	Y	42.08	2.17	5.15	1.55	0.1	6.66	19.99	1.1	5.52
	Z	43.73	1.6	3.67	1.67	0.02	1.45	8.79	1.75	19.87
	XZ	45.42	0.25	0.55	1.67	0.01	0.83	9.98	0.42	4.19
	YZ	41.32	0.28	0.67	1.54	0.02	1.52	12.39	0.98	7.95
	All	43.05	1.86	4.33	1.61	0.07	4.49	14.4	5.24	36.43
MJF	X	42.81	0.87	2.04	1.59	0.03	1.93	13.07	1.9	14.57
	Y	36.76	3.38	9.19	1.51	0.04	2.87	17.23	3.49	20.28
	Z	43.42	4.88	11.24	1.73	0.04	2.4	8.8	4.68	53.12
	XZ	44.39	1.95	4.39	1.6	0.07	4.39	11.27	1.22	10.82
	YZ	44.22	2.77	6.27	1.65	0.08	5.04	11.96	1.12	9.41
	All	42.32	4.06	9.6	1.61	0.09	5.69	12.46	3.86	30.93

**Table 11:** Descriptive statistic of flexural strength $\sigma_{f}$, flexural modulus $E_{f}$, and flexural elongation $\varepsilon_{f}$ of SLS and MJF specimens.

Method	Orientation	$\sigma_{f}$, MPa			$E_{f}$, GPa			$\varepsilon_{f}$, %		
		Mean	Std	CV	Mean	Std	CV	Mean	Std	CV
SLS	X	61.88	0.85	1.38	1.47	0.03	2.05	7.89	0.25	3.17
	Y	60.96	1.35	2.21	1.47	0.06	4.39	7.28	0.87	12.01
	Z	60.3	1.92	3.18	1.54	0.08	5.52	7.92	0.42	5.35
	XZ	60.52	0.42	0.69	1.43	0.01	0.53	7.77	0.26	3.36
	YZ	62.63	0.57	0.91	1.46	0.02	1.4	8.03	0.32	4
	All	61.26	1.4	2.28	1.47	0.06	4.03	7.78	0.52	6.72
MJF	X	62.77	6.56	10.46	1.65	0.12	7.26	7.12	0.99	13.91
	Y	68.01	1.65	2.43	1.69	0.04	2.22	7.43	0.39	5.19
	Z	67.65	1.85	2.74	1.63	0.05	2.87	7.87	0.35	4.49
	XZ	65.31	2.36	3.62	1.62	0.04	2.5	7.04	0.79	11.26
	YZ	64.17	3.01	4.68	1.52	0.05	3.33	7.99	0.3	3.81
	All	65.73	3.74	5.69	1.62	0.08	5	7.48	0.69	9.2

**Table 12:** Results (*p*-value) of an ANOVA for repeated measures investigating the effect of manufacturing method and model orientation on the parameter values obtained in the flexural test.

Effect	$\sigma_{f}$	$E_{f}$	$\varepsilon_{f}$
Method	<0.001	<0.001	0.058
Orientation—SLS	0.009	0.015	0.103
Orientation—MJF	0.082	0.004	0.062

**Table 13:** Descriptive statistic of impact strength $R_{e}$ of SLS and MJF specimens.

Method	Orientation	$R_{e}$, kJ/m^2^		
		Mean	Std	CV
SLS	X	5.96	0.62	10.33
	Y	6.96	1.37	19.64
	Z	2.4	0.58	24.36
	XZ	2.35	0.09	4.02
	YZ	2.21	0.07	3.04
	All	3.98	2.19	55.15
MJF	X	2.63	0.64	24.26
	Y	2.68	0.62	23.15
	Z	3.44	0.34	9.79
	XZ	2.73	0.56	20.46
	YZ	2.73	0.56	20.46
	All	2.84	0.6	21.04

**Table 14:** Production capacity comparison of the EOS P396 with the HP MJF 5200 [106].

	Optimized for Mechanical Properties		Optimized for Economic Efficiency	
Parameter	EOS P396	HP MJF 5200	EOS P396	HP MJF 5200
Powder cost	63 €/kg	35 €/kg	63 €/kg	35 €/kg
Number of parts in working chamber	27	21	45	33
Number of orthosis sets	9	7	15	11
Nesting density	1.96%	1.82%	3.7%	3.37%
Refresh rate	50%	80%	50%	80%
Bulk density of powder	0.45 g/cm^3^	0.425 g/cm^3^	0.45 g/cm^3^	0.425 g/cm^3^
Density of parts	0.93 g/cm^3^	1.01 g/cm^3^	0.93 g/cm^3^	1.01 g/cm^3^
Total part volume	1357.95 cm^3^	745.7 cm^3^	2263.4 g/cm^3^	1471.9 g/cm^3^
Mass of powder used	8790 g	8046 g	16,600 g	14,898 g
Weight of sintered components	1262.9 g	753.1 g	2104.9 g	1486.6 g
Total powder cost	EUR 581.4	EUR 349.5	EUR 1098	EUR 647.1
Total powder cost per job	EUR 1154.7	EUR 1000.9	EUR 1671.3	EUR 1498.5
Preparation	2 h	0.75 h	2 h	0.75 h
Build time/job	20.38 h	11.6 h	20.38 h	11.6 h
Cool down time/job	10 h	10 h	10 h	10 h
Setup time and unpacking time	2.5 h	1 h	2.5 h	1 h
Total manufacturing time	34.88 h	23.35 h	34.88 h	23.35 h

**Table 15:** Production scenario for EOS P396.

Production Scenario for EOS P396 for 9/15 Orthosis Sets					
Monday 9:00	Tuesday 9:00	Wednesday 9:00	Thursday 9:00	Friday 9:00	Saturday 9:00
JOB 1					
		JOB 2			
				JOB 3	

**Table 16:** Production scenario for HP MJF 5200.

Production Scenario for HP MJF 5200 for 7/11 Orthosis Sets				
Monday 9:00	Tuesday 9:00	Wednesday 9:00	Thursday 9:00	Friday 9:00
JOB 1				
	JOB 2			
		JOB 3		
			JOB 4	
				JOB 5

**Table 17:** Production capacity comparison of the EOS P396 with the HP MJF 5200 based on hospital case study (263 forearm injuries in July; 214 injuries in December—summary 477 forearm injuries).

	Optimized for Mechanical Properties		Optimized for Economic Efficiency	
Parameter	EOS P396	HP MJF 5200	EOS P396	HP MJF 5200
Number of parts in working chamber	27	21	45	33
Number of orthosis sets	9	7	15	11
Number of work chambers required to complete the need for orthosis in July	30	38	18	24
Number of work chambers required to complete the need for orthosis in December	24	31	15	20
Total number of possible technological operations to be performed during one week	3	5	3	5
Total number of weeks to complete the job for July and December	18	14	11	9
Total cost per job	EUR 1154.7	EUR 1000.9	EUR 1671.3	EUR 1498.5
Total production cost	EUR 20,784.8	EUR 14,012.6	EUR 18,384.3	EUR 13,486.8

## Data Availability

Data are contained within the article.

## Abbreviations

The following abbreviations are used in this manuscript:

SLS	Selective Laser Sintering
HP MJF	HP MultiJet Fusion
PBF	Powder Bed Fusion
FFF	Fused Filament Fabrication
AM	Additive Manufacturing
DFAM	Design for Additive Manufacturing
MVR	Volumetric Melt Flow
CAM	Computer Aided Manufacturing
PA12, PA2200	Polyamide 12
FEM	Finite Element Method
Std	Standard Deviation
CV	Coefficient of Variation
$\sigma_{t}$	ultimate tensile strength
$E_{t}$	tensile modulus
$\varepsilon_{t}$	tensile elongation at break
$\sigma_{f}$	flexural strength (at maximum load)
$E_{f}$	flexural modulus
$\varepsilon_{f}$	flexural elongation (at maximum load)
$R_{e}$	impact strength
